# Signal detection theory applied to giant pandas: Do pandas go out of their way to make sure their scent marks are found?

**DOI:** 10.1002/ece3.10517

**Published:** 2023-09-12

**Authors:** Yue Wang, Ronald R. Swaisgood, Wei Wei, Hong Zhou, Feiyun Yuan, Mingsheng Hong, Han Han, Zejun Zhang

**Affiliations:** ^1^ Key Laboratory of Southwest China Wildlife Resources Conservation (Ministry of Education) China West Normal University Nanchong China; ^2^ Liziping Giant Panda's Ecology and Conservation Observation and Research Station of Sichuan Province Nanchong China; ^3^ Conservation Science and Wildlife Health San Diego Zoo Wildlife Alliance Escondido California USA; ^4^ Sichuan Tibetan Area Expressway Co., Ltd Chengdu China; ^5^ Sichuan LuShi Expressway Co., Ltd Chengdu China

**Keywords:** *Ailuropoda melanoleuca*, chemical communication, conservation implication, scent‐marking

## Abstract

Inter‐animal communication allows signals released by an animal to be perceived by others. Scent‐marking is the primary mode of such communication in giant pandas (*Ailuropoda melanoleuca*). Signal detection theory propounds that animals choose the substrate and location of their scent marks so that the signals released are transmitted more widely and last longer. We believe that pandas trade‐off scent‐marking because they are an energetically marginal species and it is costly to generate and mark chemical signals. Existing studies only indicate where pandas mark more frequently, but their selection preferences remain unknown. This study investigates whether the marking behavior of pandas is consistent with signal detection theory. Feces count, reflecting habitat use intensity, was combined with mark count to determine the selection preference for marking. The results showed that pandas preferred to mark ridges with animal trails and that most marked tree species were locally dominant. In addition, marked plots and species were selected for lower energy consumption and a higher chance of being detected. Over 90% of the marks used were the longest‐surviving anogenital gland secretion marks, and over 80% of the marks were oriented toward animal trails. Our research demonstrates that pandas go out of their way to make sure their marks are found. This study not only sheds light on the mechanisms of scent‐marking by pandas but also guides us toward more precise conservation of the panda habitat.

## Graphical abstract

This study provides a more rigorous approach to the study of scent marking in giant pandas and concludes that it is indeed consistent with signal detection theory.

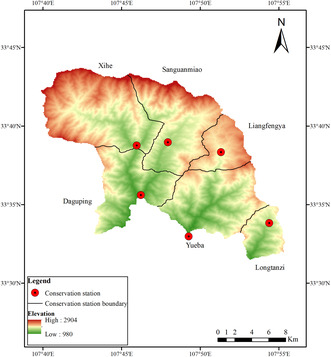

## 1 INTRODUCTION

Animal communication refers to the process by which an individual sends signals to other individuals and the individual receiving the signals responds to them (Johnson, 1973). Chemical communication, with characteristics such as specificity and delay, is one of the main modes of communication in most mammals (Elwell et al., 2021), as it is independent of light and can still propagate when the signal releaser is away from the chemical signal (Campbell‐Palmer & Rosell, 2011; Wyatt, 2014). Scent‐marking, the deposition of secretions from exocrine scent glands or by scent in urine and feces at key substrate locations (Roth et al., 2022), is a common form of source of the chemical signal in mammals (Gosling & Roberts, 2001). The main vehicle for transmitting information in mammalian chemical communication is the chemical pheromone, which includes complex information, such as individual identity characteristics, sex, age, reproductive status, social status, and kinship (Brennan & Kendrick, 2006; Ferrero & Liberles, 2010; Johansson & Jones, 2007).

The signal detection theory propounds that animals select scent‐mark signal deposit substrates, resulting in a wider range and longer retention of the released signal (Alberts, 1992; Claase et al., 2022). This choice of signal deposit substrate is widespread in mammalian chemotaxis, such as the spotted hyena (*Crocuta crocuta*; Gorman, 1990), leopard (*Panthera pardus*; Rafiq et al., 2020), and African wild dog (*Lycaon pictus*; Claase et al., 2022). These animals use urine and feces, among other substances, for chemical communication, and deposit them in frequently visited, specific sites. However, we need to be aware that both chemical signal generation and marking are energy‐consuming (Gosling et al., 2000); and after marking, the animal needs to visit the site periodically to observe and update the signal marks to maintain the continued validity of the signal, a process that requires a significant amount of time and energy (Clapham et al., 2013; Roberts & Gosling, 2001). The economic constraints associated with travel and time costs of chemical signaling across an animal's entire home range preclude range‐wide scent signal saturation, forcing animals to be strategic about selecting scent deposition sites.

Giant pandas (*Ailuropoda melanoleuca*) are typically solitary mammals that rarely come into direct contact with other individuals except for the rutting season when they form gatherings (Nie, Swaisgood, Zhang, Hu, et al., 2012; Schaller, 1985). Their long‐term life in the dense bamboo forests has led to relative vision degradation (Wei, Hu, et al., 2015), so information exchange between these giant panda individuals in the wild relies mainly on olfaction and hearing (Hu et al., 1985). Auditory communication refers to communication between individuals by acoustic signals (Barker, 2023) and it occurs during the mating aggregation period in the breeding season (Charlton et al., 2009) or during casual contact between individuals in the non‐breeding season (Schaller, 1985). Scent‐marking behavior thus becomes a major mode of communication for wild pandas (Swaisgood et al., 1999; Wei, Swaisgood, et al., 2015). Pandas communicate chemically mainly by depositing anogenital gland secretion (AGS) and urine to transfer information between conspecifics (Hu et al., 1985). Furthermore, this indirect communication between pandas maintains the community structure of this solitary species (Zhou et al., 2022).

However, the cost of generating chemical signals is too high for an energetically marginal species like the panda, which uses urine and AGS as chemical communication signals rather than feces (Nie, Swaisgood, Zhang, Hu, et al., 2012; Swaisgood et al., 2020). Both urine and feces are metabolic byproducts and therefore consume less energy, whereas AGS is produced by specialized glands and has a high‐fat content (Hagey & Macdonald, 2003), further exacerbating energy expenditure. Yet, there is an irreplaceable role for AGS in inter‐individual communication in pandas. Some of these volatile compounds are only present during the mating season and their relative abundance varies with the breeding season; thus, these compounds are directly related to panda reproduction (Zhou, Nie, Hu, et al., 2019).

Existing studies show that pandas mostly scent‐mark ridges and select suitable substrate material based on these marks (Nie, Swaisgood, Zhang, Hu, et al., 2012). However, this result may stem from the fact that they happen to be there rather than a selection preference. Thus, it is crucial to investigate whether pandas scent‐mark more frequently than expected at a given location as an indication of selection preference. In this regard, feces were collected during our sample line survey, and fecal density was used to determine panda habitat use intensity. Because pandas defecate nearly 50 times per day and do not appear to use feces for communication (Nie, Swaisgood, Zhang, Hu, et al., 2012), their choice of defecation location is random, depending only on where they are located when they defecate.

This study illustrates the preferences of pandas when scent‐marking and proposes the use of feces count to reflect habitat use intensity. We hypothesized that pandas trade‐off scent‐marking by finding the most likely location for their signal to be detected by their conspecifics while using as little energy as possible, which is consistent with signal detection theory. This study can precisely inform the conservation of pandas and the protection of their habitats.

## 2 MATERIALS AND METHODS

### 2.1 Study sites

**FIGURE 1:**
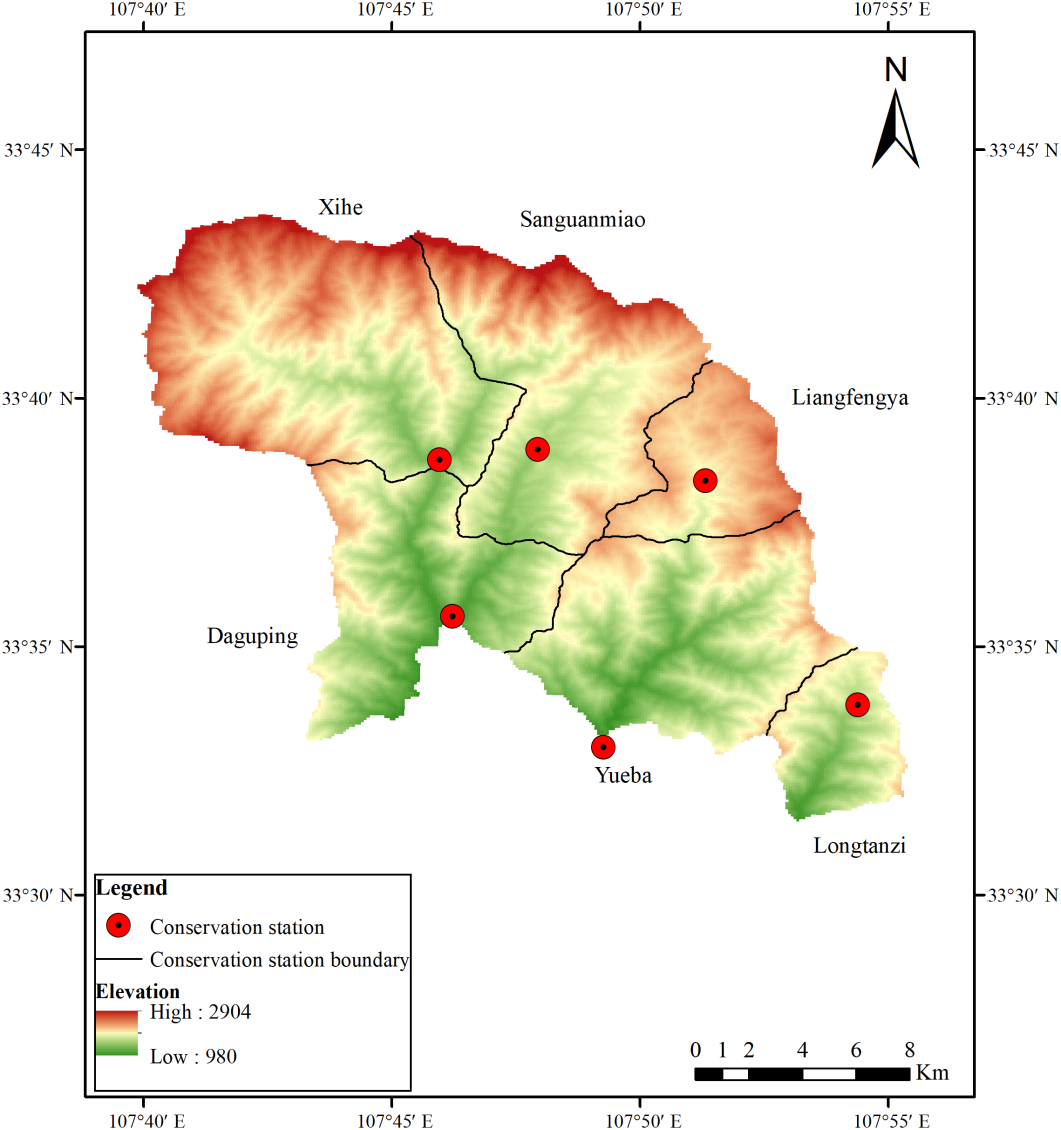
Map of the Foping National Nature Reserve.

This study was conducted in Shaanxi Foping National Nature Reserve (Figure 1) in the Qinling Mountains, with a geographical range between 107°41′ and 107°55′ E and 33°33′ and 33°46′ N. The total area is about 293 km^2^, and the elevation ranges from 980 to 2904 m. According to *The Fourth National Survey Report on the Giant Panda in China*, about 67 wild pandas were present in the reserve (State Forestry Administration, 2015). The six conservation stations in the reserve are Liangfengya, Sanguanmiao, Xihe, Daguping, Longtanzi, and Yueba. Among these, Sanguanmiao and Xihe are the core areas of the reserve and are rich in flora and fauna. In addition to the panda, there are a range of national key protected animals such as the takin (*Budorcas taxicolor*), forest musk deer (*Moschus berezovskii*), and leopard.

### 2.2 Field data collection

We established a sample line near Sanguanmiao, the core area of the reserve, where the panda density is known to be high and thus with a high number of panda scent marks (Nie, Swaisgood, Zhang, Hu, et al., 2012). Six types of sample lines were established along three types of terrains—ridge, valley, and slope (Table 1): 12, 7, and 3 samples with animal trails, and 11, 7, and 4 without animal trails; the former as experimental samples and the latter as control samples. The total length of the 22 experimental lines was 18.02 km, and the total length of the 22 control lines was 16.37 km. The length of the transect was determined by the natural topography. Animal trails were identifiable wildlife trails caused by repeated trips, evident by worn appearance, compacted soil, and lack of vegetation (Nie, Swaisgood, Zhang, Hu, et al., 2012).

**TABLE 1:** Topographic definitions of transects.

Topography	Definition
Ridge	Narrow raised area, typically 2–5 m wide, with steep slopes on either side
Valley	Low and narrow areas between two hills, often with a water source
Slope	Surface higher at one end or side than at the other, a rising or falling level

The width of the sample line was 4 m, that is, 2 m on each side of the road, and marked trees and feces were easily detected within this range. A modified method of Nie, Swaisgood, Zhang, Hu, et al. (2012) was used for data collection (Nie, Swaisgood, Zhang, Hu, et al., 2012). We recorded the number of panda feces found along the sample line, species and location of all trees with a diameter at breast height (DBH) greater than 5 cm (termed available trees), and several characteristics that may affect the selectivity of panda marks (Table 2; Zhou, Nie, Swaisgood, et al., 2019). We described the preference index as (marked trees/km)/(feces/km). Tree species marked by pandas were counted, and their marking frequency was classified into three categories—low frequency (1–5 marks), medium frequency (6–10 marks), and high frequency (>10 marks).

**TABLE 2:** Main variables measured for marked or control trees and sites.

Variables	Definition and description
Mark type	Anogenital gland secretion mark, bite mark, urine mark, and scratch mark
Mark orientation	Whether the mark is face to, parallel to, or back to trail
DBH (cm)	Tree trunk diameter at 1.5 m above ground level
Roughness	Divided into four categories: (1) smooth, (2) relatively smooth, (3) relatively rough, and (4) rough
SDT (cm)	Straight line distance from the tree to the center of the trail
Topography	Whether the site is located in ridge, valley, or slope
Slope	Slope of the marked tree or the tree in the middle of control sites
Slope aspect	Aspect of the slope of the marked tree or the tree in the middle of control sites
Vegetation density	Tree, shrub, and bamboo density in the sites
Vegetation cover	Tree, shrub, and bamboo cover in the sites

For trees with scent marks, additional characteristics, such as mark type and orientation, were recorded (termed marked trees). Urine marks cause a green bark with a musky smell, and AGS marks turn the bark brown. To determine if marked trees were repeatedly marked, each marked tree in the sample line was monitored every other week. The musky smell of urine marks disappeared after about 5 days, which allowed us to determine if the same area was repeatedly marked. For AGS marks, a small piece of bark was cut in the middle of the marked area, leaving out the light‐colored bark underneath, and it was monitored to see if it was again covered with AGS marks to determine if it was repeatedly marked (Zhou, Nie, Hu, et al., 2019; Zhou, Nie, Swaisgood, et al., 2019).

For each scent‐marked tree, where feasible, we established a 5 × 5 m plot centered on the tree and measured the variables of vegetation density, vegetation cover, slope, and slope aspect (Table 2). Afterward, a control sample was established by walking 200 m along the sample line and measuring the same indicators. If marked trees were located within the control sample, we repositioned the sample to the nearest area not containing marked trees to form a better control (Nie, Swaisgood, Zhang, Hu, et al., 2012).

### 2.3 Data analysis

The Mann–Whitney *U* test and one‐way ANOVA were used to determine the extent to which the presence or absence of the sample line of the animal trail influenced the preference of pandas for marking, based on whether the independent variables conformed to a normal distribution and the homogeneity of variance. The Kolmogorov‐Smirnov test was used to determine whether the independent variables conformed to the normal distribution. The Kruskal–Wallis test was used for the degree of preference for different terrains. In addition, the Mann–Whitney *U* test and chi‐square test were used to determine whether individual variables significantly differed between the marked and control samples, and between the marked and control trees. Subsequently, Pearson correlation analysis was used to determine the correlation among the variables, and after eliminating the significantly correlated variables, stepwise regression was used to build the best model to determine which variable had the greatest effect on the selection of marking sites by pandas. In this study, differences were statistically significant at *p* < .05, and all tests were two‐tailed. Statistical analyses were carried out by using IBM SPSS Statistics 27.0.1 software.

## 3 RESULTS

The total length of the 22 experimental lines was 18.02 km, and the total length of the 22 control lines was 16.37 km. A total of 152 scent‐marked trees were found in the experimental lines and five in the control lines. A total of 790 panda feces were found in the experimental lines and 498 in the control lines (Table S1).

### 3.1 Terrains preferred by pandas for marking

**FIGURE 2:**
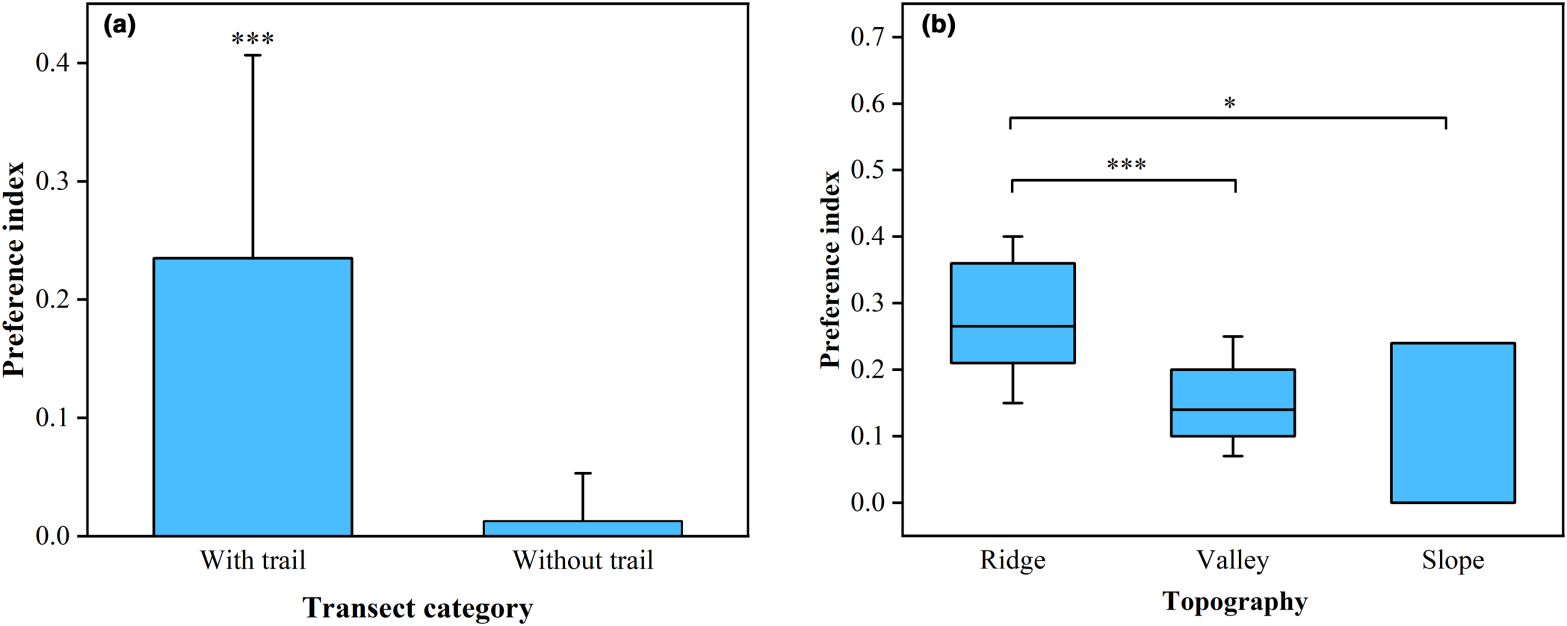
Comparison of preference index of (a) transects with and without animal trails (Mann–Whitney *U* test = 33.00, *p* < .001) and (b) different topographies with trails (Kruskal–Wallis test, *χ* ^2^ = 9.74, *p* = .008).

A significant difference was found in the degree of marking preference between experimental and control lines, with pandas preferring to scent‐mark lines with animal trails (Mann–Whitney *U* test = 33.00, *p* < .001; Figure 2a). The terrain preference also significantly differed, with pandas preferring to scent‐mark ridges, followed by valleys, and finally slopes (Kruskal–Wallis test, *χ* ^2^ = 9.74, *p* = .008; Figure 2b). The preferred location for scent‐marking by pandas was ridges with animal trails (preference index = 0.24 ± 0.08), whereas slopes without animal trails were not selected (preference index = 0; Table S1).

### 3.2 Tree species preferred by pandas for marking

There were 16 species of low frequency, four species of medium frequency, and three species of high frequency. The three species with high frequency are Badung oak (*Quercus engleriana*), Yaupon pine (*Pinus tabulaeformis*), and Huashan pine (*P. armandii*; Table S2).

### 3.3 Microhabitat differences between marked and control plots

A total of 157 scent‐marked trees were found in the sample line survey, but due to topography, slope, etc., not every location of marked trees was suitable for plots; thus, we made 144 marked plots corresponding to 114 control plots. Bamboo density (*U* = 5474.00, *p* < .001), bamboo cover (*U* = 5905.50, *p* < .001), and slope (*U* = 6328.50, *p* < .001) were lower in marked plots than in control plots. However, tree density (*U* = 4731.00, *p* < .001) and tree cover (*U* = 5729.00, *p* < .001) were higher in marked plots than in control plots. Shrub density (*U* = 7523.50, *p* = .226) and shrub cover (*U* = 7500.00, *p* = .130) were not significantly different between both groups (Tables S3, S4). After excluding significantly correlated variables identified from the Pearson correlation analysis, parameters including tree density, bamboo density, slope, and tree and shrub cover were entered into a stepwise logistic regression equation for further analysis; the results showed that tree cover was the most explained variable for whether pandas chose the location for scent‐marking or not (Table 3), namely, pandas preferred to scent‐mark sites with high tree cover.

**TABLE 3:** Variables entered into the stepwise logistic regression equation between marked and control sites.

Variables	Non‐standard regression coefficient		Standard regression coefficient	*t*	Sig.
	*B*	SE	*β*		
Tree density	0.048	0.018	0.204	2.614	*p* = .009
Bamboo density	−0.015	0.004	−0.166	−3.366	*p* < .001
Slope	−0.097	0.030	−0.234	−3.192	*p* = .002
Tree cover	0.169	0.027	0.739	6.145	*p* < .001
Shrub cover	0.085	0.037	0.176	2.272	*p* = .024

### 3.4 Microhabitat differences between marked and control trees

Our survey identified 157 marked trees, corresponding to 441 unmarked control trees, with significant differences in bark roughness between both groups (*χ* ^2^ = 19.31, *p* < .001). DBH was greater in scent‐marked trees than in control trees, but the difference was not significant (*U* = 34,473.00, *p* = .079). Straight line distance from the tree to the center of the trail (SDT) was significantly shorter in marked trees than in control trees (*U* = 11,791.50, *p* < .001; Tables S3, S5). After excluding significantly correlated variables identified from the Pearson correlation analysis, the remaining parameters (i.e., roughness, DBH, and SDT) were entered into the stepwise logistic regression equation; the results showed that roughness explained the highest degree of tree species selection by pandas for scent‐marking (Table 4), namely, pandas preferred scent‐marking trees with high roughness.

**TABLE 4:** Variables entered into the logistic regression equation between marked and unmarked trees.

Variables	Non‐standard regression coefficient		Standard regression coefficient	*t*	Sig.
	*B*	SE	*β*		
Roughness	0.132	0.012	0.673	10.637	*p* < .001
DBH (cm)	0.004	0.001	0.156	2.977	*p* = .003
SDT (cm)	−0.002	<0.001	−0.407	−7.807	*p* < .001

### 3.5 Microhabitat differences between repeatedly and singly marked trees

We found 157 scent‐marked trees, of which 139 were marked multiple times, accounting for 88.3% of all marked trees, and 18 were marked only once, accounting for 11.7% of all marked trees (Figure S1a). One‐way ANOVA was performed on factors that might affect the frequency of marking, and no significant differences were found between roughness (*p* = .65), DBH (*p* = .82), and SDT (*p* = .70; Table S6).

### 3.6 Panda mark types and orientations

Mark type and orientation used for scent‐marking were recorded. AGS marks, comprising 144 of the 157 scent‐marked trees, were the most frequent marks, accounting for 91.7% of all marked trees. Scratch marks, comprising four of the 157 scent‐marked trees, were the least frequent marks, accounting for 2.5% of all marked trees, and all of them were accompanied with AGS marks (Figure S1b). In mark orientation, face to trail, comprising 128 of the 157 scent‐marked trees, was the most frequent (81.5%), whereas back to trail, with six trees, was the least frequent (3.8%), and multidirectional marks were accompanied with face to trail marks (Figure S1c).

## 4 DISCUSSION

Panda scent‐marking behavior is an interesting study, but most previous studies have neglected to combine panda habitat use intensity to derive a one‐sided picture of panda marking preferences (Hou et al., 2021; Nie, Swaisgood, Zhang, Hu, et al., 2012). Our study used feces count as a corroboration of habitat use intensity and combined it with scent mark count, contributing to our understanding of scent‐marking preferences of pandas. This approach can also be valuable for the study of scent‐marking preferences in other species.

Our results showed that pandas preferred to scent‐mark ridges with animal trails, and we know by the feces density of the sample line that this is indeed the area with the highest panda habitat use intensity as well (Table S1). The presence of many animal trails on ridges also makes them important paths for the movement of pandas (Bai et al., 2020). However, some areas without animal trails still had high fecal densities, but the preference index for pandas to choose these areas for scent‐marking was low. Pandas might have chosen these locations for scent marking not because they happen to be there but because they need such terrains to maximize signal propagation efficiency. They might have also chosen these trails, which are often visited by other animals, as a way of indicating their presence to the other species. For instance, brown bears (*Ursus arctos*) and several other carnivore and ungulate species investigate bear smell on rubbing trees (Penteriani et al., 2023). Our results can thus guide the protection of panda habitats more precisely.

Tree species most frequently scent‐marked by pandas were Badung oak, Yaupon pine, and Huashan pine, which are dominant species with a wide local distribution (Ming et al., 1999). We believe that they chose the dominant species for marking because these trees may be more conspicuous and take less time and energy to be found (Gonzalez‐Bernardo et al., 2021). In addition to wide distribution, their high marking frequency may be because they are well located for signal consistency and can become scent stations over time (Hu et al., 1985), and they are often located along animal trails on mountain ridges (Nie, Swaisgood, Zhang, Hu, et al., 2012). Markings on these scent stations commonly used by pandas also increased the likelihood of their information being detected by signal receivers. Zhou, Nie, Hu, et al. (2019) demonstrated that both volatile and non‐volatile compounds in AGS marks were not significantly degraded within 2 weeks, indicating that pandas visit and update marks frequently (Zhou, Nie, Hu, et al., 2019). Thus, depositing marks in better‐located scent stations also reduced the energetic cost of returning and updating marks. Future work could increase research on scent stations and explore their microhabitats and conditions, such as light, that may affect the persistence and transmission of marks to increase the conservation of habitats where potential scent stations exist.

Roughness and SDT of trees marked by pandas significantly differed from control trees, where pandas usually do not scent‐mark smooth trunks, which reduced the persistence of marking. A rougher bark also increases the evaporation surface of the marks to increase spread efficiency (Zhou, Nie, Swaisgood, et al., 2019). SDT of marked trees was significantly shorter than that of control trees because the closer the distance to the animal trail, the more likely it is to be detected by the signal recipient, and because when marking here, pandas can travel shorter distances and save energy; habitats of this type are also frequently the core area of the panda habitat (Bai et al., 2020).

Compared with control plots, marked plots had lower bamboo density, bamboo cover, and slope, whereas the tree density and cover were higher. High bamboo density and cover make walking in bamboo forests more difficult for pandas, whereas low bamboo density allows them to obtain sufficient nutrition while reducing energy expenditure to traverse bamboo forests (Wei, Nie, et al., 2015). As for the lower slope of the marked samples, the reason is that the gentle slope is a suitable habitat preferred by pandas (Hu et al., 1985). A lower slope ensures that pandas complete scent‐marking in a relatively steady posture (Hou et al., 2021), while walking on a gentle slope reduces energy expenditure. The high tree density and cover in marked samples were due to the fact that the more trees there are, the greater the chance of trees with larger DBH, and trees with larger DBH are more likely to form tree dens for pandas to breed and nurse their cubs (Wei et al., 2018; Zhang et al., 2011). In addition, the higher the tree density, the more concealed the habitat, and thus, the less likely the pandas are to be detected by natural predators (Hu et al., 1985).

The number of trees marked repeatedly (i.e., repeatedly marked trees) was much higher than trees marked only once (i.e., singly marked trees), but significant differences were not detected between the variables of either. We speculate that the more prominent position and wider field of view of repeatedly marked trees may also increase the chances of the signal being found by both other pandas and other species. Alternatively, these locations may be associated with other essential resources in the home range of the individual, making them more frequent in specific areas (Gonzalez‐Bernardo et al., 2021). To disseminate personal information more consistently, the mark type most used by pandas is AGS marks, which often remain in the natural environment for more than 3 months because of their low volatility (Hagey & Macdonald, 2003; Swaisgood et al., 2004). In contrast, urine marks contain more volatile substances than AGS marks with a shorter retention time in the environment, and are often used to convey the estrus status of females (Zhou, Nie, Swaisgood, et al., 2019) and competitive ability of males (White et al., 2002).

Scratch marks were accompanied with AGS marks; thus, scratch marks alone do not convey information. However, scratch marks cannot be considered a byproduct of chemical marking and their exact implications need to be further explored (Penteriani et al., 2023). Interestingly, some marked trees had only bite marks, and these marked trees were more numerous than those with only urine marks, which was not found in previous studies. We assume that the role of scratch marks is correlated with mark height; if the mark is higher, the individual is larger and more competitive, similar to AGS and urine marks (White et al., 2002); however, the specific role of scratch marks needs to be further investigated. McGuire and Bemis (2017) found that body size affects the frequency of marking in a study of domestic dogs (*Canis lupus familiaris*; Mcguire & Bemis, 2017). However, from studies of pandas, we know that only body size reflects their competitive ability (Nie, Swaisgood, Zhang, Liu, & Wei, 2012), but whether it affects the frequency of marking deserves further investigation.

Statistical data on mark orientation revealed that most marks were face to trail, which is because orientation toward the animal trail increases the chance of the mark being detected by a conspecific or being easier to reach in order to save energy. For marks that were back to trail, we predicted possible scent counter‐marking behavior, which is one of the main responses of animals when they encounter the scent of competitors of the same species (Johnson, 1973). However, before counter‐marking, they evaluate individuals already marked, because they are not as competitive as the former and want to mark a superior position on the marked tree; thus, they mark the back (Müller & Manser, 2008). However, the exact reason needs to be confirmed by future studies.

Footpad scent communication has been found in the fellow bear species brown bear (Sergiel et al., 2017) and polar bear (*U. maritimus*; Owen et al., 2015), and most bear species have large home ranges, where releasing scent while walking is an effective form of intraspecific communication (Penteriani & Melletti, 2020). An interesting question is whether pandas similarly use footpad scent for communication. In this study, sites with pitted panda tracks were monitored (Figure S2). Unfortunately, we did not find other pandas observing and sniffing tracks in our follow‐up observations. This may be due to our small sample size or the fact that we did not specifically focus on this direction, and we hope to continue this study to confirm this possibility.

The frequency of marking in pandas (Nie, Swaisgood, Zhang, Hu, et al., 2012) and the compound content of AGS marks (Zhou, Nie, Hu, et al., 2019) significantly differ between the sexes. Crowned lemurs (*Eulemur coronatus*) also show similar sex differences in scent‐marking behaviors (Elwell et al., 2021). Zhou, Nie, Hu, et al. (2019) detected large differences in the composition and content of compounds in AGS marks between captive and wild pandas (Zhou, Nie, Hu, et al., 2019), which may also be a reason for the low reproductive ability of captive pandas. Similar findings were reported in captive female gentle lemur (*Hapalemur alaotrensis*), whose perianal gland scent marks reflect their fertility (Fontani et al., 2022). Future research on scent marks of pandas in the field should be carried out to clarify which compounds affect the estrus and reproduction of pandas, and applied to captive pandas to improve their reproductive success. Direct detection of compounds contained in the scent marks of pandas in the field is difficult, but the use of PerkinElmer's Torion® gas chromatograph/mass spectrometer can make this possible (Poirier et al., 2021). This is a tremendous step forward for the survival and reproduction of panda populations. The scent‐marking behavior of the female sloth bear (*Melursus ursinus*) is influenced by the presence or absence of males, and it is worth exploring whether the same behavior occurs in pandas (Khadpekar et al., 2021).

Current research on chemical communication has still not kept up with acoustic communication (Chen & Wiens, 2020). Because of the construction of the Giant Panda National Park, human facilities will inevitably be built in the living environment of pandas. Human facilities can affect the scent‐marking behaviors of domestic felines (Krofel et al., 2017), and scent‐marking of pandas should be continuously studied to detect whether their marking behavior will change because of the influence of human facilities (e.g., assessing the impact range size in human facilities before their construction and then locating them as far away as possible from the core panda habitat), so that panda habitats can be conserved more precisely. Furthermore, habitat fragmentation, a major threat to panda survival, has been impeding communication between panda populations (Kang, 2022), and the construction of ecological corridors in conjunction with habitats marked by panda preferences can predictably increase corridor use and thereby mitigate this threat, which is one of the implications of our findings.

It has to be acknowledged that our study has its limitations, such as a small sample size, but given the environment in which pandas live, it is difficult to collect large amounts of data in the wild; thus, our results are likely to be preliminary. Expanding the scope of the study area and harvesting data from different seasons to obtain more accurate results are promising directions for future research, which is worth the effort and can help researchers to conserve panda populations and their habitats in a more precise manner.

## 5 CONCLUSION

Previous studies of scent‐marking in wild pandas have only identified areas of high marking density (Nie, Swaisgood, Zhang, Liu, & Wei, 2012; Zhou, Nie, Swaisgood, et al., 2019), but this study combined panda habitat use intensity to derive a more rigorous picture of their marking preferences, although this result is only preliminary because of the limitation of a small sample size. Such results are not surprising as pandas are energetically marginal species limited by energy deficits in many aspects of their lives (Hu et al., 1985). Thus, there is no doubt that they are particularly sensitive to the energy‐consuming behavior of generating and depositing chemical signals. Although our findings are basic exploratory work, they can help researchers to conserve panda populations and their habitats in a more precise manner. In addition, referring to such habitats when building ecological corridors can facilitate exchanges between pandas and increase the effectiveness of the corridors.

## AUTHOR CONTRIBUTIONS

**Yue Wang:** Data curation (equal); formal analysis (equal); writing – original draft (equal). **Ronald R. Swaisgood:** Writing – review and editing (equal). **Wei Wei:** Resources (equal); writing – review and editing (equal). **Hong Zhou:** Writing – review and editing (equal). **Feiyun Yuan:** Funding acquisition (equal). **Mingsheng Hong:** Writing – review and editing (equal). **Han Han:** Supervision (equal); writing – review and editing (equal). **Zejun Zhang:** Supervision (equal); writing – review and editing (equal).

## CONFLICT OF INTEREST STATEMENT

The authors declare no conflict of interest.

## Supporting information


Appendix S1.

## Data Availability

Data that are used to calculate are openly available in Dryad at https://doi.org/10.5061/dryad.nzs7h44x5. All data used in the analyses described in this study can be found in the Supporting Information.
