# Supplementary material for: Signal detection theory applied to giant pandas: Do pandas go out of their way to make sure their scent marks are found?
Source: Ecol Evol. 2023 Sep 12;13(9):e10517. doi: 10.1002/ece3.10517 (PMC10495809; doi:10.1002/ece3.10517)
Supplement: Supplementary file 1 — Appendix S1. [file ECE3-13-e10517-s001.docx]

**Appendix**


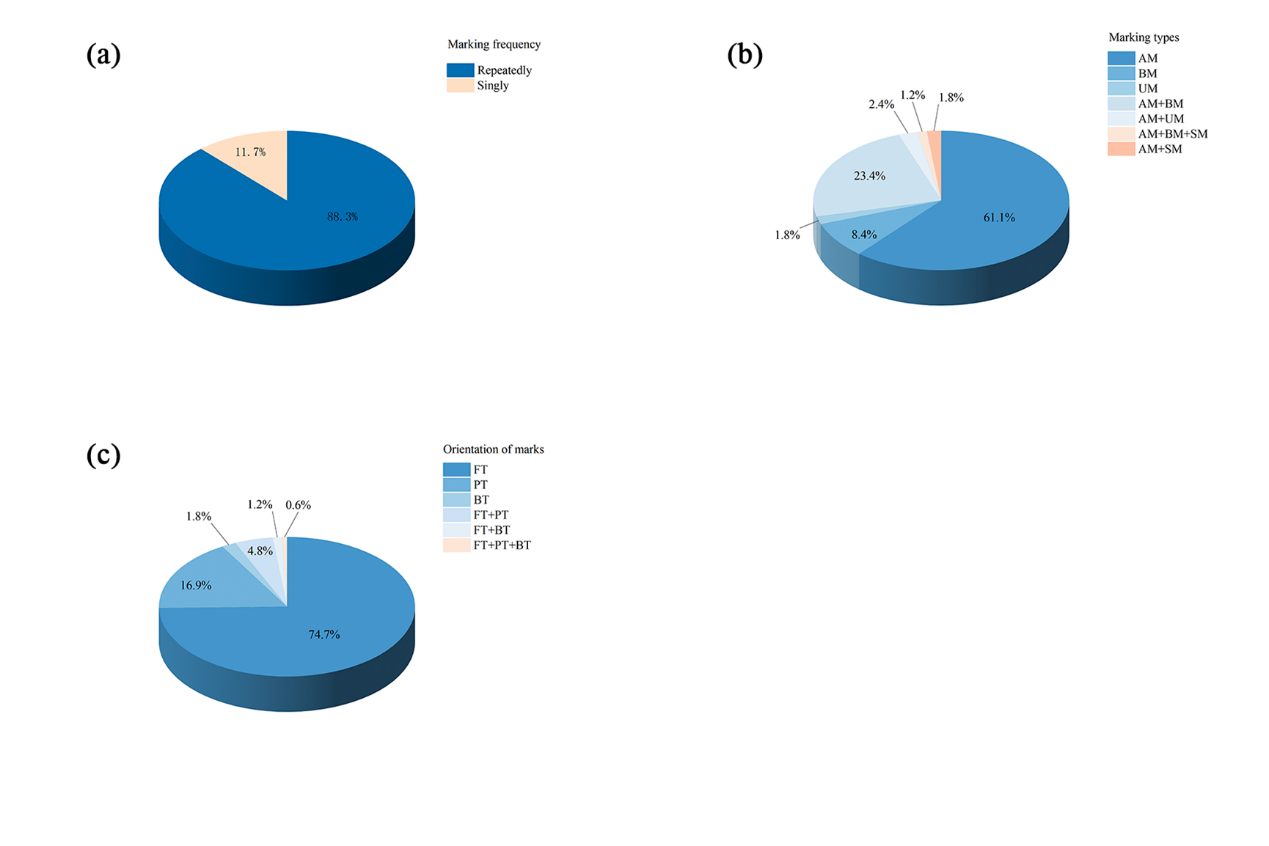


Figure S1 (a) Frequency of scent-marked trees; (b) frequency of panda mark types (AM = anogenital gland secretion mark, BM = bite mark, UM = urine mark, SM = scratch mark); (c) frequency of panda mark orientations (FT = face to trail, PT = parallel to trail, BT = back to trail).


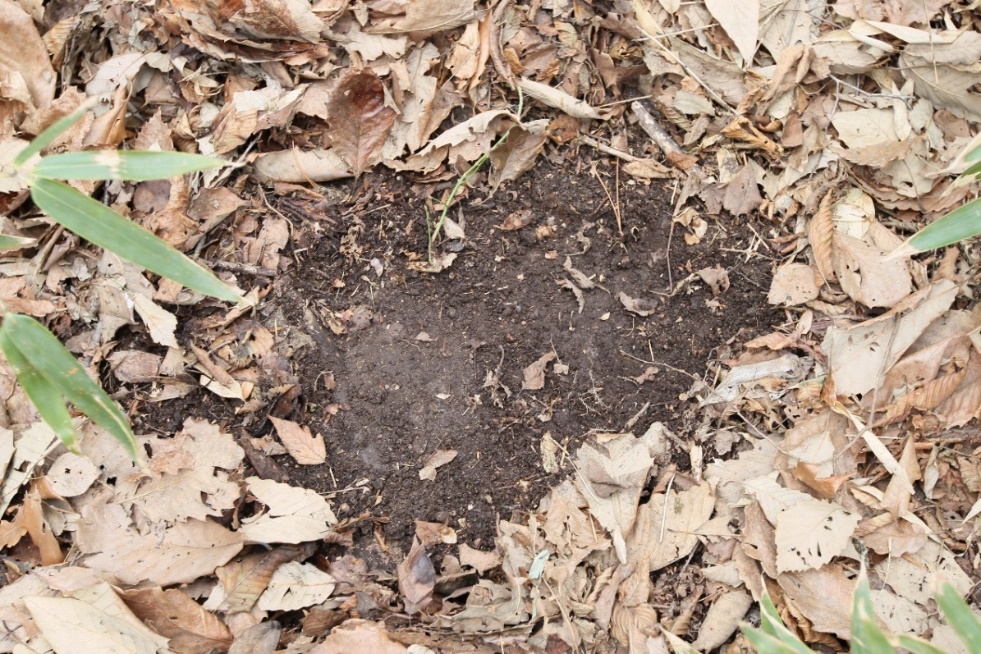


Figure S2 Panda plowing behavior and footprints.

Table S1 Summary of surveyed transects at Sanguanmiao District, Foping National Nature Reserve

| Type | Trail transects | | | No-trail transects | | |
| --- | --- | --- | --- | --- | --- | --- |
|  | Valley | Slope | Ridge | Valley | Slope | Ridge |
| Number of transects | 7 | 3 | 12 | 7 | 4 | 11 |
| Total length of transects (km) | 6.85 | 1.45 | 9.72 | 6.5 | 1.75 | 8.12 |
| Number of feces | 250 | 37 | 503 | 119 | 90 | 289 |
| Feces/km | 36.50 | 25.52 | 51.75 | 18.31 | 51.43 | 35.59 |
| Number of marked trees | 30 | 5 | 117 | 2 | 0 | 3 |
| Marked trees/km | 4.38 | 3.45 | 12.04 | 0.31 | 0.00 | 0.37 |
| Preference index* | 0.15 ± 0.06 | 0.12 ± 0.17 | 0.33 ± 0.17 | 0.004 ± 0.007 | 0.00 | 0.023 ± 0.06 |

*: Preference index = (marked trees/km)/(feces/km).

Table S2 Tree species selected for marking by wild pandas and their marking frequency

| Marking frequency | Marked tree species (scientific name) |
| --- | --- |
| Low (1–5) | *Magnolia denudata, Toxicodendron vernicifluum, Cotoneaster submultiflorus, Prunus davidiana, Quercus spinosa, Elaeagnus henryi, Abies fargesii, Juglans cathayensis, Caripinus turczaninowii, Fraxinus mandschurica, Alchornea davidii, Pterocarya hupehensis, Betula albosinensis, Ligustrum obtusifolium, Cornus hemsleyi, Tilia chinensis* |
| Medium (6–10) | *Castanea mollissima, Prunus tomentosa, Tsuga chinensis, Populus cathayana* |
| High (>10) | *Q. engleriana, Pinus tabulaeformis,* *P. armandii* |

Table S3 Kolmogorov-Smirnov test for variables in marked and control sites and trees

| Variables | Mean SD | | *P* |
| --- | --- | --- | --- |
|  |  |  |  |
| Bamboo density | 5.41 | 6.18 | *P* < 0.001 |
| Bamboo cover | 1.55 | 0.83 | *P* < 0.001 |
| Tree density | 2.61 | 1.89 | *P* < 0.001 |
| Tree cover | 3.18 | 0.78 | *P* < 0.001 |
| Shrub density | 1.19 | 1.43 | *P* < 0.001 |
| Shrub cover | 1.38 | 0.69 | *P* < 0.001 |
| Slope  Roughness  DBH  SDT | 1.59  2.52  2.70  89.21 | 0.84  0.97  5.27  62.86 | *P* < 0.001  *P* < 0.001  *P* < 0.001  *P* < 0.001 |

Note: The *p*-value is less than 0.05, which does not conform to the homogeneity of variance.

Table S4 Mann–Whitney *U* test for variables between marked and control sites

| Variables | Mean ± SD or frequency | | *U* (*P*) |
| --- | --- | --- | --- |
|  | Marked sites | Control sites |  |
| Bamboo density  (culms/m^2^) | 3.71 ± 4.02 | 7.56 ± 7.63 | *U* = 5474.00 (*P* < 0.001) |
| Bamboo cover (%) | 0–25%: 108; 26–50%: 25; 51–75%: 9; 76–100%: 2 | 0–25%: 56; 26–50%: 31; 51–75%: 20; 76–100%: 7 | *U* = 5905.50 (*P* < 0.001) |
| Tree density  (culms/plot) | 3.20 ± 1.77 | 1.87 ± 1.76 | *U* = 4731.00 (*P* < 0.001) |
| Tree cover (%) | 0–25%: 2; 26–50%: 12; 51–75%: 59; 76–100%: 71 | 0–25%: 4; 26–50%; 30; 51–75%: 50; 76–100%: 30 | *U* = 5729.00 (*P* < 0.001) |
| Shrub density  (culms/plot) | 1.22 ± 1.32 | 1.15 ± 1.58 | *U* = 7523.50 (*P* = 0.226) |
| Shrub cover (%) | 0–25%: 98; 26–50%: 33; 51–75%: 11; 76–100%: 2 | 0–25%: 88; 26–50%: 17; 51–75%: 7; 76–100%: 2 | *U* = 7500.00 (*P* = 0.130) |
| Slope (°) | 0–10°: 99; 10–20°: 34; 20–30°: 7; >30°: 4 | 0–10°: 55; 10–20°: 32; 20–30°: 20; >30°: 7 | *U* = 6328.50 (*P* < 0.001) |

Table S5 Comparison of variables between marked and unmarked trees

| Variables | Mean ± SD or frequency | | *U* or *χ^2^* (*P*) |
| --- | --- | --- | --- |
|  | Marked trees | Unmarked trees |  |
| Roughness | smooth (1): 22; relatively smooth (2): 65; relatively rough (3): 51; rough (4): 19 | smooth (1): 87; relatively smooth (2): 97; relatively rough (3): 185; rough (4): 72 | *χ^2^* = 19.31 (*P* < 0.001) |
| DBH (cm) | 15.78 ± 12.00 | 15.28 ± 13.26 | *U* = 34473.00 (*P* = 0.079) |
| SDT (cm) | 41.59 ± 22.75 | 107.97 ± 63.73 | *U* = 11791.50 (*P* < 0.001) |

Table S6 One-way ANOVA for variables between repeatedly and singly marked trees

| Variables | Mean ± SD | | *P* |
| --- | --- | --- | --- |
|  | Repeatedly | Singly |  |
| Roughness | 2.43 ± 0.91 | 2.53 ± 0.84 | *P* = 0.65 |
| DBH (cm) | 16.18 ± 12.46 | 15.47 ± 10.56 | *P* = 0.82 |
| SDT (cm） | 41.99 ± 23.93 | 44.21 ± 16.85 | *P* = 0.70 |
